# Favorable Changes in Basic Functional Status and Mobility After Participation in a Community-Based Day Center Program for Older Adults: A Pre–Post Study of Two Independent Annual Cohorts in Chile

**DOI:** 10.3390/geriatrics11040082

**Published:** 2026-07-07

**Authors:** Armando Cifuentes-Amigo, Claudia Fica, Ignacio Salas, Nacim Molina, Diego Arauna, Eduardo Fuentes, Iván Palomo

**Affiliations:** 1 Thrombosis and Healthy Aging Research Center, Department of Clinical Biochemistry and Immunohematology, Faculty of Health Sciences, Interuniversity Center for Healthy Aging (CIES), Interuniversity Network of Healthy Aging in Latin America and the Caribbean (RIES-LAC), Vitalis-Longevity Center, Universidad de Talca, Talca 3640000, Chile; 2 National Service for Senior Citizens (SENAMA), Region of Maule, Talca 3460000, Chile

**Keywords:** healthy ageing, day care center, older people

## Abstract

**Introduction**: Community-based day center programs may support healthy ageing by promoting functional ability, mental well-being, and social participation among older adults, but real-world evidence from Latin America remains limited. **Objective**: We aimed to examine changes in functional status, mental health, and quality of life among older adults participating in the CEDIAM program in the Maule Region of Chile in 2022 and 2023. Methods: Pre–post observational study using routinely collected data from 15 CEDIAM centers. The 2022 and 2023 datasets were analyzed as independent cohorts. Functional status was assessed with the Barthel Index, the Lawton and Brody scale, and the Timed Up and Go test; mental health with the Mini-Mental State Examination and the 15-item Geriatric Depression Scale; and quality of life with the EuroQol-5D visual analogue scale. Paired comparisons, category-transition analyses, and multivariable logistic regression models of improvement were performed. **Results**: Baseline samples included 894 participants in 2022 and 897 in 2023. In 2022, all continuous outcomes improved significantly (all *p* ≤ 0.001). In 2023, the Barthel Index, the Timed Up and Go test, and the Geriatric Depression Scale improved (all *p* < 0.0001), and the EuroQol-5D visual analogue scale also improved (*p* < 0.01), whereas the Lawton and Brody scale (*p* = 0.204) and the Mini-Mental State Examination (*p* = 0.725) did not. Category-transition analyses showed significant improvements in basic activities of daily living and mobility in both cohorts (both *p* < 0.001), while significant categorical changes in instrumental activities of daily living, global cognition, depressive symptoms, and self-rated quality of life were observed only in 2022 (all *p* ≤ 0.01). Rural residence was associated with higher odds of improvement in basic activities of daily living (OR 1.62, 95% CI 1.17–2.25; *p* = 0.004), whereas age ≥75 years was associated with lower odds of improvement in depressive symptoms (OR 0.56, 95% CI 0.41–0.76; *p* < 0.001) and self-rated quality of life (OR 0.65, 95% CI 0.45–0.94; *p* = 0.023). **Conclusions**: Participation in CEDIAM was associated with favorable changes, particularly in basic functional status and mobility, although responses varied across outcomes and participant subgroups.

## 1. Introduction

Population ageing is transforming health and social care systems worldwide and has become one of the most important public health challenges of the 21st century [1,2]. In this context, healthy ageing has increasingly been understood not only as survival into older age, but as the ability to maintain functional capacity, autonomy, psychological well-being, and participation in community life. This broader perspective is especially relevant in countries such as Chile, where demographic ageing has progressed rapidly over recent decades and now poses major challenges for health systems, social support, and long-term care policies [3,4,5,6]. This approach is also consistent with the contemporary framework of healthy ageing proposed by the World Health Organization, which emphasizes the maintenance of functional ability as a central goal in later life [7].

Within this scenario, community-based care models for older adults have gained increasing attention as potential strategies to support ageing in place, preserve independence, and reduce the need for institutional care [8]. Among these, day care centers (DCCs) for older people may provide opportunities to improve social participation, mental health, physical functioning, and quality of life [9,10]. However, the literature also shows substantial heterogeneity in the structure, aims, and intensity of these services, which makes it difficult to compare findings across studies and to draw firm conclusions regarding effectiveness [9]. Recent reviews suggest that day center-based interventions may offer benefits in rehabilitation, activity promotion, and social participation, but they also emphasize marked variability in intervention components, professionals involved, and outcomes assessed [11]. Likewise, evidence on psychosocial interventions delivered in day care services highlights potential benefits, while underscoring the diversity and complexity of these models [12]. Consistent with this, recent work has emphasized that community participation is central to the design and operation of effective day care center models for older adults, particularly in middle-income and mixed urban–rural settings [13].

Evidence from comparable community-based day programs, although heterogeneous, points to several recurring benefits. Regular day care attendance has been associated with lower mortality among community-dwelling frail older people [8], and reviews of day centers for older people report favorable influences on mental health, social contact, and quality of life, while noting that these services are predominantly used by older women with declining health and from lower socio-economic backgrounds [10]. At the same time, this body of work consistently highlights persistent evidence gaps, including the limited regulation and standardization of these services and the difficulty of evaluating outcomes across heterogeneous program models [9,10]. In Chile, evidence remains particularly scarce: a previous evaluation of a multidimensional program delivered in senior day centers assessed physical and mental health, functional status, and quality of life, but was limited by a small sample and a relatively short, medium-term follow-up [14]. Larger, real-world evaluations across multiple centers and outcome domains are therefore needed to clarify the contribution of day center programs to healthy ageing in the region.

In Chile, one of the main public strategies aimed at promoting healthy ageing is the *Centros Diurnos del Adulto Mayor* (Community Day Centers for Older Adults; CEDIAM) program, launched in 2013 by the National Service for Older Adults (SENAMA) as part of the country’s policy response to population ageing [15]. The program is designed to support community-dwelling older adults aged 60 years and over in maintaining autonomy, strengthening social inclusion, and promoting active participation in their local environment, while also offering preventive support to caregivers [16]. CEDIAM centers provide structured activities focused on personal care, social integration, and community engagement, and older adults are expected to attend at least three times per week for a minimum of six months [15]. Given its expansion across the country and its relevance within the Chilean social and health policy landscape, evaluating the real-world performance of this program is important from both a gerontological and a public health perspective.

Despite its policy relevance, the effectiveness of CEDIAM has been only scarcely evaluated. Previous studies in Chile have generally involved smaller samples, shorter follow-up periods, or narrower outcome assessment [14,15,16]. Moreover, the extent to which participation in CEDIAM is associated with changes across multiple domains of healthy ageing remains insufficiently characterized, particularly in real-world program settings. This evidence gap is particularly relevant in Latin America, where rapid population ageing coexists with high levels of functional dependency and limited long-term care infrastructure, yet real-world evaluations of community-based programs remain scarce [17]. Therefore, the present study aimed to examine changes in functional status, mental health, and quality of life among older adults participating in CEDIAM centers in the Maule Region of Chile during 2022 to 2023.

## 2. Methods

### 2.1. Study Design and Data Source

This was a pre–post observational study based on routinely collected data from the CEDIAM program in the Maule Region, Chile. Data were obtained from the 15 CEDIAM centers operating in the region during 2022 and 2023 and were provided by the National Service for Older Adults (SENAMA). Because no individual participant was assessed in both calendar years, the datasets from 2022 and 2023 were considered independent cohorts.

### 2.2. The CEDIAM Program

The CEDIAM program is a community-based day center initiative targeting community-dwelling older adults (≥60 years) with mild dependence, and in some cases mild cognitive impairment or mild depressive symptoms. Each center has capacity for 30, 60, or 90 participants, who are expected to attend at least three times per week, following an individualized comprehensive care plan defined for a six-month period that may be extended. Services are organized around three domains: a personal domain, comprising individualized care for the older person, caregiver-oriented actions, and general services; a social domain, consisting of workshops aimed at strengthening ties among participants and with the community; and a community domain, involving activities that promote participation, self-management, and community engagement. Activities are delivered by a multidisciplinary team—which may include social workers, physiotherapists, occupational therapists, and psychologists—and typically combine physical, cognitive, psychosocial, educational, and recreational components. Each center is operated by a different public or non-profit organization selected through a competitive public funding process, and the distribution of professional hours and activities is adjusted to local needs and resources. Consequently, although all centers share a common programmatic framework defined by SENAMA, the precise intensity, composition, and delivery of activities may vary across centers—a source of variability that, together with the use of routinely collected data, should be considered when interpreting the findings.

### 2.3. Participants

**Figure 1:**
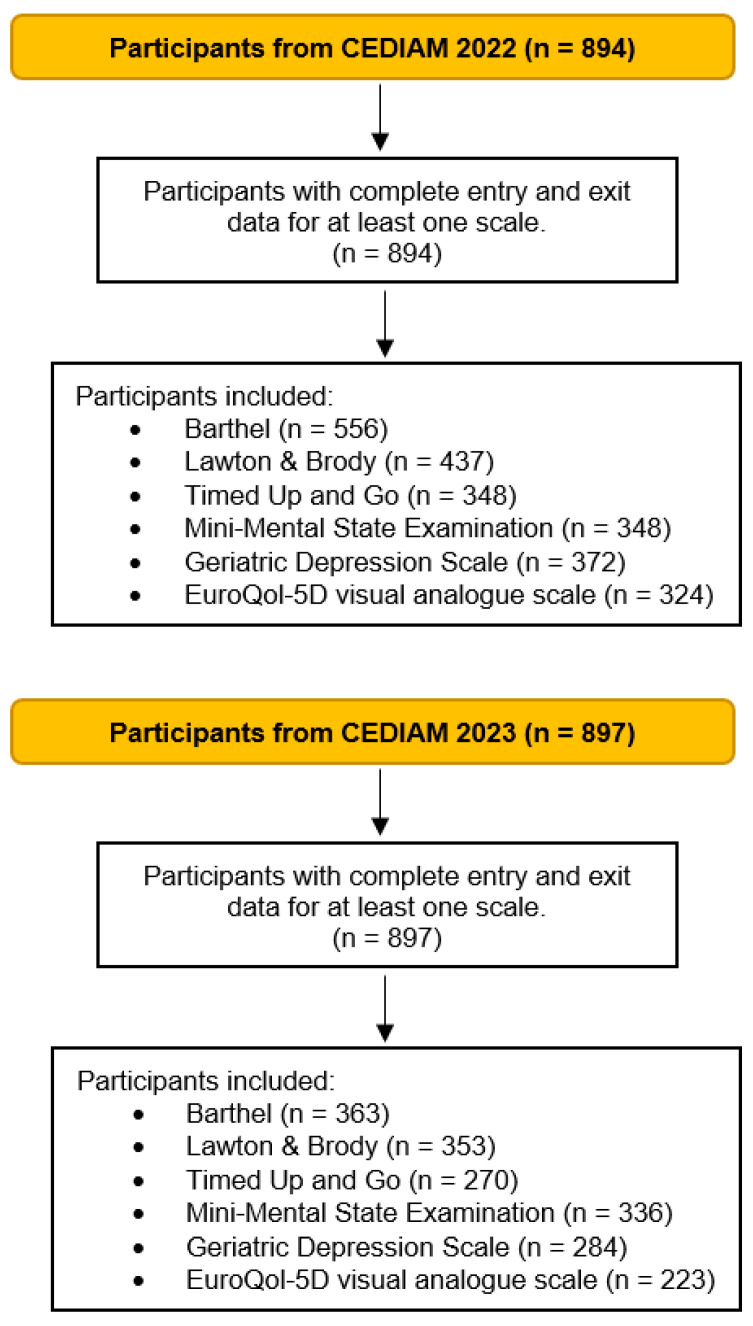
Flowchart of Participant Selection for the CEDIAM Evaluation Study (2022–2023). Sample size varies by scale according to availability of complete entry and exit assessment.

Participants were eligible for inclusion if they had completed the required entry and exit assessments established by the CEDIAM program for at least one of the outcomes evaluated in this study. Records were excluded when information was missing, inconsistent, or clearly erroneous for the specific instrument under analysis. Because completeness varied across instruments, analyses were conducted separately for each scale using only participants with paired entry–exit data for that specific outcome. Therefore, the analytical sample differed across instruments, and no integrated complete-case analysis including all three domains simultaneously was performed. The participant selection process and the resulting scale-specific analytical samples are summarized in Figure 1.

### 2.4. Geriatric Assessment and Study Outcomes

The geriatric assessment covered three domains: functional status, mental health, and quality of life. All assessments were conducted by trained CEDIAM staff following standardized program procedures.

### 2.5. Functional Status

Functional status was assessed using three instruments. The Barthel Index (BI) was used to evaluate basic activities of daily living, with scores ranging from 0 to 100 points, where higher scores indicate greater independence. For categorical analyses, BI scores were classified as independent (100 points), mild dependence (>60 and <100 points), and moderate dependence (≤60 points) [18,19]. Instrumental activities of daily living were assessed using the Lawton and Brody scale (L&B), with scores ranging from 0 to 8 points and higher values indicating greater independence. For categorical analyses, scores were classified as independent (8 points), mild dependence (6–7 points), moderate dependence (4–5 points), severe dependence (2–3 points), and total dependence (0–1 point) [19,20]. Mobility and fall risk were assessed using the Timed Up and Go test (TUG), which measures the time required to stand up from a chair, walk three meters, turn, return, and sit down again. Participants performed the test without physical assistance. For categorical analyses, TUG performance was classified as no risk of falls (<10 s) and risk of falls (≥10 s) [19,21].

### 2.6. Mental Health

Mental health was assessed using the Mini-Mental State Examination (MMSE) and the 15-item Geriatric Depression Scale (GDS-15). The MMSE evaluates global cognitive function on a 0–30-point scale, with higher scores indicating better performance. Cognitive status was categorized as no cognitive impairment (>27 points), mild cognitive impairment (22–26 points), and suspected dementia (≤21 points) [19,22]. Depressive symptoms were assessed with the GDS-15, which includes 15 yes/no items and yields a total score ranging from 0 to 15 points. Scores were categorized as no depression (0–5 points), mild depression (6–9 points), and moderate-to-severe depression (≥10 points) [19,23].

### 2.7. Quality of Life

Quality of life was assessed using the EuroQol-5D visual analogue scale (EQ-5D), in which participants rate their perceived health status from 0 (worst imaginable health) to 100 (best imaginable health). For categorical analyses, scores were grouped as excellent/very good/good (≥60 points) and fair/poor (≤59 points) [24,25].

### 2.8. Definition of Improvement

For secondary analyses, improvement was defined as any increase in score between entry and exit for BI, L&B, MMSE, and EQ-5D, and as any reduction in time or score for TUG and GDS-15, respectively.

### 2.9. Covariates

The following participant characteristics were included in multivariable analyses: residence (urban/rural), sex (men/women), age group (<75 years/≥75 years), multimorbidity (<2/≥2 chronic conditions), educational level (low education: primary school completed or less; higher education: above primary school), arterial hypertension (yes/no), and type 2 diabetes mellitus (yes/no).

### 2.10. Statistical Analysis

Analyses were performed using IBM SPSS Statistics version 23.0 (IBM Corp., Armonk, NY, USA) and GraphPad Prism version 8. Descriptive data are presented as median and interquartile range (IQR) for continuous variables and as number and percentage for categorical variables. Baseline comparisons between the 2022 and 2023 cohorts were performed using the Mann–Whitney U test for continuous variables and the chi-square test for categorical variables. Within each cohort, pre–post changes in continuous scale scores were evaluated using the Wilcoxon signed-rank test. Changes in paired categorical classifications were analyzed using the McNemar test for outcomes with two categories and the McNemar–Bowker test for outcomes with more than two categories. When global tests were statistically significant, post hoc pairwise comparisons with Bonferroni correction were applied. All pre–post comparisons, category-transition analyses, and multivariable logistic regression models were performed using scale-specific paired samples. Thus, participants contributed data only to the analyses for which complete entry and exit assessments were available. No imputation of missing data was performed.

To identify sociodemographic and clinical factors associated with improvement in each outcome, multivariable binary logistic regression models were fitted separately for each scale (BI, L&B, TUG, MMSE, GDS-15, and EQ-5D). For each instrument, the dependent variable was coded as 1 for participants who improved between entry and exit and 0 for those who did not improve, according to the predefined criteria for each scale. Independent variables were entered as binary covariates: residence (urban = 0, rural = 1), sex (men = 0, women = 1), age group (<75 years = 0, ≥75 years = 1), multimorbidity (<2 = 0, ≥2 chronic conditions = 1), educational level (higher education = 0, low education = 1), arterial hypertension (no = 0, yes = 1), and type 2 diabetes mellitus (no = 0, yes = 1). Because participants belonged exclusively to one cohort and no individual was evaluated in both years, analyses were conducted per outcome without repeated-measures adjustment by year. Adjusted odds ratios (ORs) and 95% confidence intervals (CIs) were estimated. Multicollinearity was assessed using variance inflation factors (VIFs), all of which were <2. A two-tailed *p*-value < 0.05 was considered statistically significant.

### 2.11. Data Authorization and Ethical Considerations

This study was based on a governmental database provided and authorized for research use by the National Service for Older Adults (SENAMA). Data were analyzed in anonymized form, and no direct contact with participants or additional data collection was performed for the present study. Because this work involved the secondary analysis of anonymized program data, with no access to identifiable personal information, no additional informed consent for participation was required for the purposes of this analysis.

## 3. Results

### 3.1. Baseline Characteristics of the 2022 and 2023 Cohorts

A total of 894 participants in 2022 and 897 in 2023 were included in the baseline description (Table 1). The median age was similar across cohorts [74 (68–78) years in 2022 and 73 (68–77.5) years in 2023; *p* = 0.081]. Women represented approximately 80% of participants in both years (80.3% in 2022 and 80.5% in 2023; *p* = 0.830). Compared with the 2022 cohort, participants in 2023 were more frequently from rural areas (27.8% vs. 19.0%; *p* < 0.0001) and more often had multimorbidity (54.1% vs. 44.6%; *p* < 0.0001). Educational level was similar between cohorts, with more than half of participants in both years having low educational attainment (60.5% in 2022 and 59.2% in 2023; *p* = 0.346).

### 3.2. Changes in Continuous Scores Between Entry and Exit

Paired sample sizes varied across instruments according to the availability of complete entry and exit assessments (Table 2 and Table 3). In the 2022 cohort, statistically significant improvements were observed in all evaluated outcomes, including BI, L&B, TUG, MMSE, GDS-15, and EQ-5D (all *p* ≤ 0.001). Paired analytical samples ranged from 324 to 556 participants in 2022 and from 223 to 363 participants in 2023, depending on the instrument. Therefore, comparisons across outcomes should be interpreted as scale-specific analyses rather than as analyses conducted on a single complete-case sample including all functional, mental health, and quality-of-life measures. In the 2023 cohort, significant improvements were observed in BI, TUG, GDS-15, and EQ-5D, whereas no significant changes were detected for L&B (*p* = 0.204) or MMSE (*p* = 0.725). Distributions of individual scores at entry and exit for both cohorts are shown in Figures S1 and S2.

### 3.3. Category Transitions Between Entry and Exit

**Figure 2:**
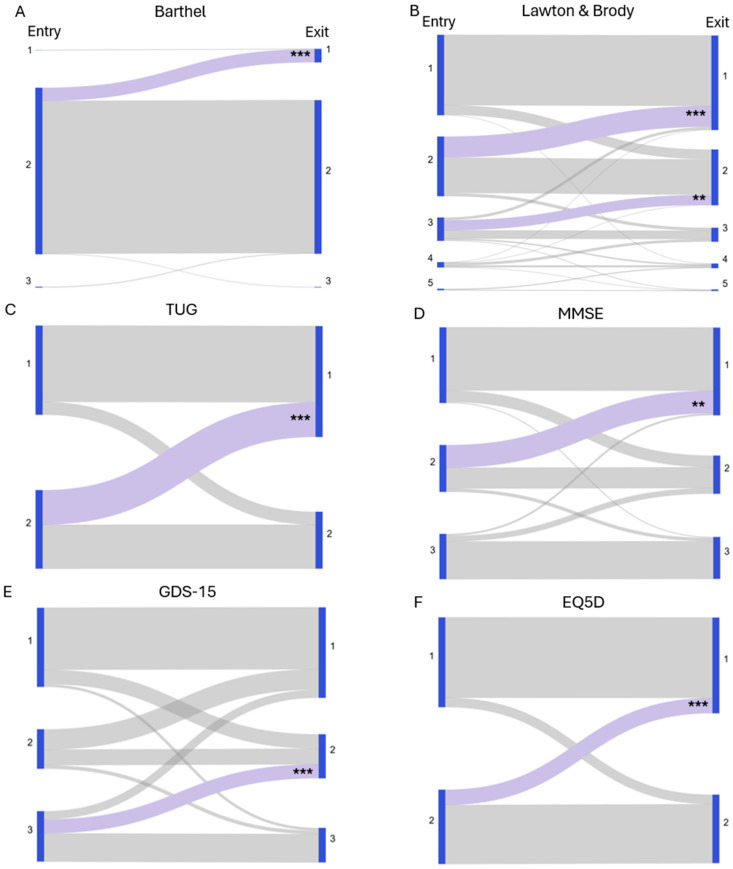
Sankey plots showing category transitions in functional status, mental health, and quality of life from entry to exit to the CEDIAM in 2022. Each panel (**A**–**F**) displays category changes for a specific scale: (**A**) Barthel Index, (**B**) Lawton & Brody, (**C**) Timed Up and Go (TUG), (**D**) Mini-Mental State Examination (MMSE), (**E**) Geriatric Depression Scale (GDS-15), and (**F**) EQ-5D. Category codes correspond to: Barthel (1 = independent, 2 = mild dependency, 3 = moderate dependency); Lawton & Brody (1 = independent, 2 = mild dependency, 3 = moderate dependency, 4 = severe dependency, 5 = total dependency); TUG (1 = no risk of falls, 2 = risk of falls); MMSE (1 = no impairment, 2 = mild impairment, 3 = suspected dementia); GDS-15 (1 = no depression, 2 = mild depression, 3 = moderate–severe depression); EQ-5D (1 = excellent/very good/good, 2 = fair/poor). Scales with two categories (TUG and EQ-5D) were evaluated using the McNemar test. Scales with three or more categories (Barthel, Lawton & Brody, MMSE, and GDS-15) were evaluated using the McNemar–Bowker test for paired categorical data. Post hoc comparisons were Bonferroni-adjusted. Purple flows indicate statistically significant transitions, while grey flows represent non-significant changes. ** *p* < 0.01, *** *p* < 0.001.

**Figure 3:**
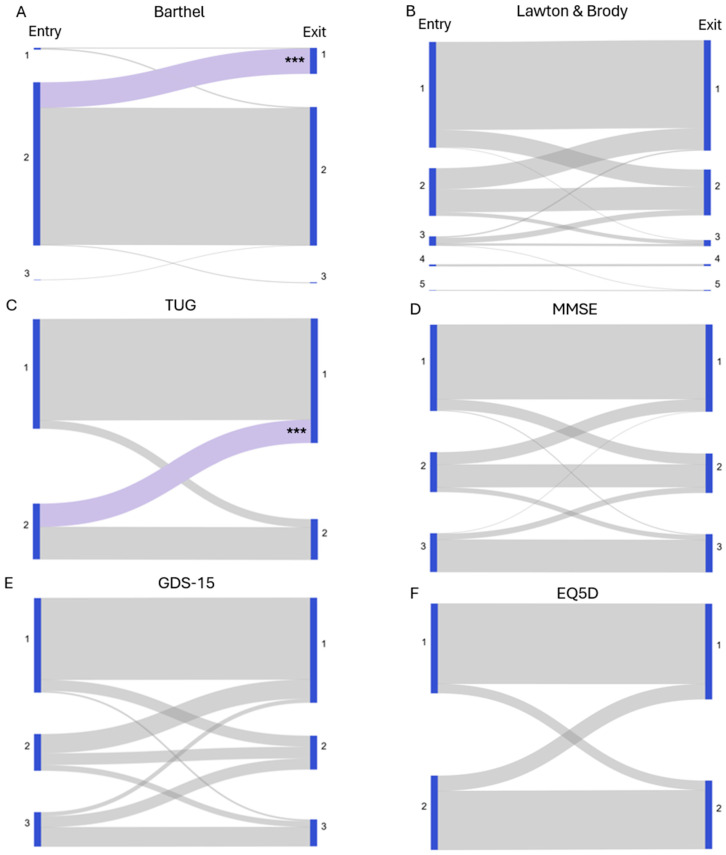
Sankey plots showing category transitions in functional status, mental health, and quality of life from entry to exit to the CEDIAM in 2023. Each panel (**A**–**F**) displays category changes for a specific scale: (**A**) Barthel Index, (**B**) Lawton & Brody, (**C**) Timed Up and Go (TUG), (**D**) Mini-Mental State Examination (MMSE), (**E**) Geriatric Depression Scale (GDS-15), and (**F**) EQ-5D. Category codes correspond to: Barthel (1 = independent, 2 = mild dependency, 3 = moderate dependency); Lawton & Brody (1 = independent, 2 = mild dependency, 3 = moderate dependency, 4 = severe dependency, 5 = total dependency); TUG (1 = no risk of falls, 2 = risk of falls); MMSE (1 = no impairment, 2 = mild impairment, 3 = suspected dementia); GDS-15 (1 = no depression, 2 = mild depression, 3 = moderate–severe depression); EQ-5D (1 = excellent/very good/good, 2 = fair/poor). Scales with two categories (TUG and EQ-5D) were evaluated using the McNemar test. Scales with three or more categories (Barthel, Lawton & Brody, MMSE, and GDS-15) were evaluated using the McNemar–Bowker test for paired categorical data. Post hoc comparisons were Bonferroni-adjusted. Purple flows indicate statistically significant transitions, while grey flows represent non-significant changes. *** *p* < 0.001.

Changes in outcome categories between entry and exit are summarized in Table S1 and illustrated in Figure 2 and Figure 3. In the functional domain, both cohorts showed a significant transition in BI categories from mild dependence to independence (*p* < 0.001). A significant reduction in fall risk according to TUG was also observed in both years, with participants moving from the “risk of falls” category to the “no risk of falls” category (*p* < 0.001). In contrast, significant upward transitions in L&B categories were observed only in the 2022 cohort, particularly from mild and moderate dependence to better functional categories (*p* < 0.001 and *p* < 0.01, respectively).

For mental health and quality of life, significant categorical changes were more evident in 2022. In that cohort, MMSE categories showed a significant shift from mild cognitive impairment to normal cognitive function (*p* < 0.01). Likewise, GDS-15 categories showed a favorable redistribution, with a reduction in moderate-to-severe depressive symptoms (*p* < 0.001). Self-rated quality of life also improved significantly in 2022, with participants more frequently moving from fair/poor to good, very good, or excellent health categories (*p* < 0.001). No significant category transitions were highlighted for MMSE, GDS-15, or EQ-5D in the 2023 cohort.

### 3.4. Factors Associated with Improvement Across Outcomes

Multivariable logistic regression models showed heterogeneity in factors associated with improvement across domains (Table 4, Table 5 and Table 6). In the BI model, rural residence was independently associated with higher odds of improvement (OR 1.62, 95% CI 1.17–2.25; *p* = 0.004). No significant associations were found for the remaining covariates. In the L&B model, female sex (OR 0.62, 95% CI 0.42–0.93; *p* = 0.019), multimorbidity (OR 0.55, 95% CI 0.36–0.84; *p* = 0.005), hypertension (OR 0.57, 95% CI 0.34–0.98; *p* = 0.041), and type 2 diabetes mellitus (OR 0.16, 95% CI 0.04–0.98; *p* = 0.017) were associated with a lower likelihood of improvement. No covariate was significantly associated with improvement in TUG performance.

In the mental health domain, hypertension was associated with lower odds of MMSE improvement (OR 0.52, 95% CI 0.29–0.94; *p* = 0.031), while age ≥ 75 years was associated with a lower likelihood of improvement in depressive symptoms according to GDS-15 (OR 0.56, 95% CI 0.41–0.76; *p* < 0.0001). Regarding quality of life, women had higher odds of improvement in EQ-5D (OR 1.62, 95% CI 1.02–2.58; *p* = 0.041), whereas participants aged ≥75 years were less likely to improve (OR 0.65, 95% CI 0.45–0.94; *p* = 0.023). No other covariates were significantly associated with improvement in MMSE, GDS-15, or EQ-5D.

## 4. Discussion

To our knowledge, this is one of the few large-scale, real-world evaluations of a publicly funded, community-based day center program for older adults in Latin America. Although previous studies have assessed day care or community-based interventions in older populations, most have relied on smaller samples, single cohorts, or a narrower set of outcomes, and evidence from Latin American settings remains particularly scarce. The present study adds to this literature in several ways: it draws on routinely collected data from a national public program (CEDIAM) as implemented in everyday practice; it includes a large sample of 1791 older adults across 15 centers and two consecutive annual cohorts; and it adopts a multidimensional approach, simultaneously examining functional status, mental health, and quality of life through six standardized instruments and complementary analytical strategies. Rather than providing a controlled efficacy estimate, this design offers a pragmatic, population-level picture of how participation in a real-world program relates to multiple domains of healthy ageing in a middle-income Latin American context.

An important finding of this study was the cohort-dependent pattern of observed change. Although the 2022 cohort showed statistically significant favorable changes across all evaluated outcomes, the 2023 cohort showed a less consistent pattern, with no significant changes in instrumental activities of daily living, as measured by the Lawton and Brody scale, or global cognitive performance, as measured by the MMSE. Differences between independent cohorts may partly explain this heterogeneity. Compared with 2022, the 2023 cohort included a higher proportion of participants with multimorbidity and had relatively high baseline scores on the Lawton and Brody and MMSE, which may have limited the ability to detect further improvement. In addition, instrumental activities of daily living and global cognitive performance are complex domains that may be less sensitive to change in a general community-based program than more proximal outcomes such as basic activities of daily living and mobility. Thus, the lack of significant change in Lawton and Brody and MMSE in 2023 should be interpreted as part of the heterogeneous, domain-specific, and cohort-dependent response pattern observed in this real-world program evaluation. Recent evidence from senior day centers further highlights substantial variation in intervention content, professional involvement, and outcomes assessed [11]. This pattern should be interpreted within the broader framework of healthy ageing, which emphasizes the maintenance of functional ability rather than the mere absence of disease [7]. In parallel, the international literature has consistently shown that day care centers for older adults are highly heterogeneous in their structure, purpose, and intensity of intervention [9,10,26].

This variability extends to the type of professionals involved, the balance between social and rehabilitative components, and the outcomes evaluated, which complicates direct comparisons across studies [9,10,11,26]. In that context, the present findings are best understood as evidence from a specific community-based public program implemented in a middle-income setting, rather than as a universal estimate of the effects of all-day center models.

In the functional domain, the most consistent findings were observed for basic activities of daily living and mobility. Improvements in BI and TUG were detected in both cohorts, suggesting that participation in CEDIAM may be associated with gains in basic autonomy and mobility-related performance. This interpretation is consistent with previous work showing that community-based or day care-related interventions may improve selected functional outcomes, particularly when they include structured activity, assessment, and follow-up [10,27,28,29,30]. Moreover, broader evidence from community-based complex interventions suggests that multidimensional care models are more likely to improve some ADL-related outcomes than broader constructs such as global quality of life [11,31].

By contrast, improvement in instrumental activities of daily living was less consistent, with significant changes observed only in one cohort and lower odds of improvement among women and participants with multimorbidity, hypertension, or diabetes. This is plausible because IADLs are more complex than basic ADLs and are likely to depend not only on physical performance, but also on cognitive demands, environmental context, social roles, and chronic disease burden [26]. Therefore, IADLs may be less responsive to a general community-based program than more proximal outcomes such as mobility or basic autonomy. This interpretation is also coherent with the broader literature showing that community-based interventions in older adults do not improve all domains equally, and that outcomes related to independence often respond differently depending on intervention complexity and baseline vulnerability [31]. This pattern is consistent with regional evidence showing that functional limitation in older adults tends to concentrate among women and those with lower educational and socioeconomic resources, underscoring that response to community-based programs may be shaped by pre-existing structural inequities [32].

The finding that rural residence was associated with greater odds of improvement in BI is noteworthy. One possible explanation is that older adults from rural areas may enter the program with greater unmet needs or lower prior access to structured supportive services, thus presenting a greater margin for functional benefit. Although this interpretation should be made cautiously, it suggests that community-based ageing programs may have particular value for populations with historically lower access to preventive or supportive care. This point is relevant in Chile, where territorial inequities remain an important consideration in the organization of services for older adults [3,6].

In the mental health domain, the findings were more heterogeneous. Continuous MMSE scores improved only in the 2022 cohort, and hypertension was associated with a lower probability of improvement in cognitive performance. This suggests that any cognitive benefit associated with participation in CEDIAM may be modest and context-dependent. Because CEDIAM is not designed as a specialized cognitive intervention and admission criteria favor participants without severe impairment, ceiling effects and limited room for cognitive gain are plausible explanations. This interpretation is also consistent with broader evidence indicating that community-based interventions may improve cognition in some settings, but that such effects are generally smaller and more variable than functional gains [31].

The pattern observed for depressive symptoms appears more favorable. In 2022, categorical transitions suggested a reduction in more severe depressive symptoms, and continuous-score analyses also supported improvement. This aligns with previous literature indicating that one of the most consistent potential benefits of day center attendance lies in the psychosocial domain, including mood, social participation, and perceived emotional support [10,27,29,30,33]. More broadly, recent evidence suggests that the mental health benefits of social participation in later life are themselves heterogeneous and may be greater in socially or socioeconomically vulnerable groups [34]. In that sense, the present findings reinforce the idea that community-based programs may influence emotional well-being not only through formal intervention content, but also through routine, connectedness, and sustained social engagement.

At the same time, the lower likelihood of GDS-15 improvement among participants aged 75 years and older suggests that depressive symptoms in older subgroups may be more strongly shaped by accumulated health burden, loss, reduced resilience, or longer-standing psychosocial adversity. Although this study cannot determine the mechanisms involved, the result is clinically relevant because it suggests that older participants may require more tailored mental health support than that provided by a general community-based program alone.

Quality of life showed a similarly heterogeneous pattern. Categorical improvement was clearer in 2022, whereas continuous-score analyses suggested a more modest but still favorable pattern. Women had higher odds of improvement, whereas participants aged 75 years and older were less likely to improve. Because EQ-5D captures self-perceived health rather than only objective function, these findings may reflect a broader and more subjective response to program participation, potentially influenced by social engagement, perceived support, or emotional adaptation. Importantly, this result is coherent with evidence indicating that improvements in quality of life are often less consistent than gains in more proximal functional outcomes in community-based interventions for older adults [31].

One of the main contributions of this study is therefore not only the documentation of pre–post changes, but the demonstration that response to a community-based ageing program is not homogeneous. The combination of paired score comparisons, category-transition analyses, and multivariable models provides a more nuanced understanding of program-associated change than a simple average pre–post comparison. From a gerontological perspective, this is important because healthy ageing trajectories are inherently diverse, and the same intervention is unlikely to affect all older adults in the same way [7]. From a programmatic perspective, it suggests that future optimization of CEDIAM may benefit from greater tailoring according to age, multimorbidity, and cardiometabolic burden.

These findings also have implications for public policy in ageing. In middle-income settings, where evidence on real-world community-based programs remains limited, this study provides relevant information on the potential contribution of day center models within broader healthy ageing strategies [35]. The results do not suggest a uniform effect across all domains; rather, they indicate that these programs may be particularly useful for supporting basic function, mobility, and selected aspects of mental well-being, while other domains may require more intensive or targeted intervention components. This interpretation is aligned with the growing international view that community-based care for older adults should be multidimensional, integrated, and adapted to varying levels of vulnerability [7,31].

It is important to note that the present study did not evaluate all formal aims of the CEDIAM program. Although CEDIAM is designed to maintain autonomy, strengthen social inclusion, promote active participation in the local environment, and provide preventive support to caregivers, the available routine database allowed us to examine only selected indicators related to functional status, mobility, mental health, and self-rated quality of life. Thus, autonomy was partially assessed through basic and instrumental activities of daily living, while mobility, cognitive status, depressive symptoms, and perceived health were assessed using standardized program instruments. However, social inclusion, community participation, caregiver support, adherence, and intervention dose were not directly measured. Therefore, the findings should be interpreted as evidence of observed changes in the assessed domains, rather than as a comprehensive evaluation of all CEDIAM objectives.

Several limitations should be considered. First, the pre–post observational design without a non-intervention comparison group limits causal inference, and the observed changes cannot be attributed exclusively to program participation. Although this design does not allow causal attribution, several features make it less likely that the observed improvements merely reflect natural temporal variation or regression to the mean. In older adults, the expected short-term trajectory of functional and cognitive status tends toward stability or decline rather than improvement, so the favorable changes observed run counter to the anticipated natural course. In addition, the improvements in basic activities of daily living and mobility were replicated across two independent annual cohorts, reducing the likelihood of a chance finding in a single sample, and the response pattern was heterogeneous and domain-specific rather than uniform, which is difficult to reconcile with a systematic artifact such as regression to the mean that would be expected to affect all outcomes similarly. Nonetheless, in the absence of a concurrent control group, a contribution of natural variation cannot be fully excluded, and the present findings should be interpreted as program-associated rather than program-caused changes. Second, because the study was based on routinely collected program data, completeness varied across instruments and analytical samples differed by scale. Consequently, the study did not allow an integrated complete-case analysis of functional status, mental health, and quality of life in the same participants. The findings should therefore be interpreted as outcome-specific pre–post changes rather than as evidence of simultaneous multidimensional improvement in all participants. Third, some instruments may have been affected by ceiling effects, particularly because the program includes relatively functional older adults at baseline. Fourth, the available database did not include detailed information on adherence, number of attended sessions, or the exact intensity and content of interventions received in each center, which limits interpretation of the mechanisms underlying improvement. In addition, although sex was included as a covariate in the multivariable models, the variable completeness across instruments did not allow a full sex-disaggregated analysis of all outcomes across both cohorts. Given the emphasis of the WHO Decade of Healthy Ageing on sex- and gender-sensitive evidence, future CEDIAM evaluations should incorporate sex-disaggregated analyses. Finally, although the study included two consecutive cohorts and 15 centers, findings correspond to one Chilean region and may not be directly generalizable to all CEDIAM settings or other national contexts.

Despite these limitations, the study has important strengths. It evaluated a large sample of community-dwelling older adults across 15 centers, included two consecutive cohorts, and examined multiple domains relevant to healthy ageing using real-world data. In addition, the combination of continuous-score analyses, category transitions, and multivariable models allowed a broader and more clinically informative understanding of participant response than prior local evaluations focused on fewer outcomes or shorter time frames.

## 5. Conclusions

Participation in the CEDIAM program was associated with favorable changes in several domains relevant to healthy ageing among community-dwelling older adults in the Maule Region of Chile, particularly in basic functional status and mobility. However, these benefits were not consistent across all outcomes, and the likelihood of improvement varied according to participant characteristics such as age, rural residence, multimorbidity, and cardiometabolic conditions. Rather than suggesting a uniform program effect, the present findings indicate that response to community-based day center participation is heterogeneous and domain-specific. From a gerontological and public health perspective, this is an important message. In ageing societies, community-based programs should not be evaluated only on whether they produce average improvement, but also on whether they help sustain functional ability in meaningful domains and whether they reach those older adults most likely to benefit. In this regard, the present study supports the relevance of day center programs as part of healthy ageing strategies in middle-income settings, especially for preserving basic autonomy, mobility, and selected aspects of emotional well-being.

At the same time, the observed heterogeneity suggests that future strengthening of CEDIAM and similar initiatives should move toward more tailored approaches. Older participants, those with multimorbidity, and those with chronic cardiometabolic conditions may require more targeted or intensive components if benefits are to extend beyond basic function toward cognition, instrumental function, and quality of life. These results also support the value of integrating program evaluation into ageing policy, so that expansion of community-based services is accompanied by better evidence on effectiveness, implementation, and equity. Future research should therefore prioritize comparative designs, longer-term follow-up, and the incorporation of additional clinically and policy-relevant outcomes, including frailty, healthcare utilization, institutionalization, and mortality. It should also examine adherence, dose of intervention, and variation across centers, which are likely to be critical for understanding why some participants improve more than others. In this way, evidence from programs such as CEDIAM can contribute not only to local service improvement, but also to the broader development of more responsive and evidence-informed ageing policies in Chile and Latin America.

## Figures and Tables

**Table 1:** Sociodemographic and clinical characteristics of participants upon entry to CEDIAM centers in 2022 and 2023.

Variables	2022 (*n* = 894)	2023 (*n* = 897)	*p*-Value
Age (years) (median ([IQR]) ^a^	74 (68–78)	73 (68–77.5)	0.081
Sex n (%) ^b^			0.830
Women	718 (80.3)	724 (80.5)	
Men	176 (19.7)	173 (19.5)	
Residence n (%) ^b^			<0.0001
Urban	724 (81)	647 (72.1)	
Rural	170 (19)	250 (27.8)	
Multimorbidity n (%) ^b^			<0.0001
<2 diseases	495 (55.4)	411 (45.9)	
≥2 diseases	399 (44.6)	486 (54.1)	
Education level n (%) ^b^			0.346
Low education (≤primary completed)	542 (60.5)	532 (59.2)	
Higher education (≥secondary uncompleted)	327 (36.6)	352 (39.1)	
Unregistered	25 (2.9)	13 (1.4)	-

Values are shown as median (IQR) or number (%). ^a^ Mann–Whitney test and ^b^ chi-square test were used for comparisons. IQR: interquartile range.

**Table 2:** Comparisons of functional status, mental health, and quality of life outcomes at entry and exit to the CEDIAM in 2022.

2022				
**Functional status**				
Scales	n (paired)	Entry (median [IQR])	Exit (median [IQR])	*p*-value
BI	556	90 (85–95)	90 (85–95)	<0.0001
L&B	437	7 (6–8)	8 (7–8)	<0.0001
TUG	348	10 (8–13)	9 (8–12)	<0.0001
**Mental health**				
MMSE	348	26 (20.3–29)	27 (21.3–30)	<0.0001
GDS-15	372	6 (3–10)	5 (3–8)	<0.0001
**Quality of life**				
EQ-5D	324	60 (12–80)	70 (14.8–80)	<0.001

Results are presented as median (IQR). Comparisons between entry and exit were performed using the Wilcoxon signed-rank test. n (paired) indicates participants with complete entry–exit assessments for each scale. BI: Barthel Index; L&B: Lawton and Brody scale; TUG: Timed Up and Go test; MMSE: Mini-Mental State Examination; GDS-15: 15-item Geriatric Depression Scale; EQ-5D: EuroQol-5D visual analogue scale; IQR: interquartile range.

**Table 3:** Comparisons of functional status, mental health, and quality of life outcomes at entry and exit to the CEDIAM in 2023.

2023				
**Functional status**				
Scales	n (paired)	Entry (median [IQR])	Exit (median [IQR])	*p*-value
BI	363	90 (85–95)	95 (90–95)	<0.0001
L&B	353	7 (7–8)	8 (7–8)	0.204
TUG	276	9 (7–11)	8 (7–10)	<0.0001
**Mental health**				
MMSE	336	27 (22–30)	27 (23–30)	0.725
GDS-15	284	5 (2–9)	4 (2–7)	<0.0001
**Quality of life**				
EQ-5D	223	60 (11–80)	60 (11–80)	<0.01

Results are presented as median (IQR). Comparisons between entry and exit were performed using the Wilcoxon signed-rank test. n (paired) indicates participants with complete entry–exit assessments for each scale. BI: Barthel Index; L&B: Lawton and Brody scale; TUG: Timed Up and Go test; MMSE: Mini-Mental State Examination; GDS-15: 15-item Geriatric Depression Scale; EQ-5D: EuroQol-5D visual analogue scale; IQR: interquartile range.

**Table 4:** Predictors of improvement in Functional status (Barthel Index, Lawton & Brody, and Timed Up and Go): multivariable logistic regression model.

	Barthel Index		Lawton & Brody		Timed Up and Go	
Covariate	OR (CI 95%)	*p*-Value	OR (CI 95%)	*p*-Value	OR (CI 95%)	*p*-Value
Residence (rural)	1.62 (1.17–2.25)	0.004	1.38 (0.94–2.04)	0.105	1.05 (0.73–1.51)	0.788
Sex (woman)	0.86 (0.61–1.20)	0.366	0.62 (0.42–0.93)	0.019	1.15 (0.78–1.68)	0.487
Age ≥ 75 years	0.98 (0.74–1.29)	0.866	1.17 (0.83–1.66)	0.360	1.02 (0.74–1.4)	0.915
Low educational level	0.87 (0.66–1.15)	0.326	1.36 (0.95–1.94)	0.097	1.29 (0.93–1.78)	0.133
Multimorbidity (≥2 diseases)	0.97 (0.70–1.35)	0.851	0.55 (0.36–0.84)	0.005	1.30 (0.82–2.06)	0.259
Hypertension	0.79 (0.51–1.21)	0.271	0.57 (0.34–0.98)	0.041	0.89 (0.51–1.54)	0.672
Type 2 diabetes mellitus	0.50 (0.18–1.11)	0.083	0.16 (0.04–0.98)	0.017	0.48 (0.17–1.34)	0.160

Reference categories: urban residence, men, age < 75 years, <2 diseases, no hypertension, no diabetes, higher education level. Outcome coded as 1 = improvement; 0 = no improvement. OR = odds ratio; CI = confidence interval.

**Table 5:** Predictors of improvement in mental health (Mini-Mental State Examination and Geriatric Depression Scale): multivariable logistic regression model.

	Mini-Mental State Examination		Geriatric Depression Scale	
Covariate	OR (CI 95%)	*p*-Value	OR (CI 95%)	*p*-Value
Residence (rural)	0.92 (0.6–1.37)	0.677	1.02 (0.71–1.47)	0.904
Sex (woman)	1.21 (0.81–1.80)	0.347	1.08 (0.74–1.57)	0.697
Age ≥ 75 years	0.99 (0.72–1.36)	0.936	0.56 (0.41–0.76)	<0.0001
Low educational level	1.34 (0.97–1.85)	0.080	0.91 (0.66–1.25)	0.569
Multimorbidity (≥2 diseases)	1.34 (0.89–2.02)	0.166	1.20 (0.79–1.81)	0.387
Hypertension	0.52 (0.29–0.94)	0.031	1.38 (0.81–2.36)	0.234
Type 2 diabetes mellitus	1.29 (0.46–3.58)	0.629	1.04 (0.35–3.07)	0.948

Reference categories: urban residence, men, age < 75 years, <2 diseases, no hypertension, no diabetes, higher educational level. Outcome coded as: 1 = improvement; 0 = no improvement. OR = odds ratio; CI = confidence interval.

**Table 6:** Predictors of improvement in quality of life (EuroQol-5D visual analogue scale): multivariable logistic regression model.

Covariate	OR (CI 95%)	*p*-Value
Residence (rural)	0.83 (0.55–1.24)	0.360
Sex (woman)	1.62 (1.02–2.58)	0.041
Age ≥ 75 years	0.65 (0.45–0.94)	0.023
Low educational level	0.93 (0.63–1.37)	0.702
Multimorbidity (≥2 diseases)	1.55 (0.93–2.60)	0.094
Hypertension	0.57 (0.31–1.06)	0.075
Type 2 diabetes mellitus	0.89 (0.31–2.55)	0.826

Reference categories: urban residence, men, age <75 years, <2 diseases, no hypertension, no diabetes, higher educational level. Outcome coded as: 1 = improvement; 0 = no improvement. OR = odds ratio; CI = confidence interval.

## Data Availability

Restrictions apply to the availability of these data. Data were obtained from the National Service for Older Adults (SENAMA), Chile, and are not publicly available due to governmental and privacy restrictions. Requests regarding access to the anonymized dataset should be directed to SENAMA and are subject to institutional authorization.

## Supplementary Materials

The following supporting information can be downloaded at: https://www.mdpi.com/article/10.3390/geriatrics11040082/s1, Table S1: Distribution of participants across functional status, mental health, and quality of life categories at entry and exit to the CEDIAM centers in 2022 and 2023; Figure S1: Boxplots showing entry and exit scores for functional status, mental health, and quality of life in 2022; Figure S2: Boxplots showing entry and exit scores for functional status, mental health, and quality of life in 2023.
