# Supplementary material for: Favorable Changes in Basic Functional Status and Mobility After Participation in a Community-Based Day Center Program for Older Adults: A Pre–Post Study of Two Independent Annual Cohorts in Chile
Source: Geriatrics (Basel). 2026 Jul 7;11(4):82. doi: 10.3390/geriatrics11040082 (PMC13397939; doi:10.3390/geriatrics11040082)
Supplement: Supplementary file 1 [file geriatrics-11-00082-s001.zip › geriatrics-4380712-supplementary.pdf]

Supplementary material

Table S1. s. Distribution of participants across functional status, mental health, and quality of life categories at entry and exit to the CEDIAM centers in 2022 and 2023.

Scales	2022		2023	
	Entry	Exit	Entry	Exit
**Barthel Index % (n)**	556		363	
Independent	0.2 (1)	8.2 (45)	1.1 (4)	15.7 (57)
Mild dependent	99.2 (552)	91.6 (509)	98.6 (358)	83.7 (304)
Moderate dependent	0.6 (3)	1.2 (2)	0.3 (1)	0.6 (2)
**Lawton & Brody % (n)**	437		353	
Independent	47.2 (206)	55.3 (242)	63.6 (226)	66.3 (236)
Mild dependent	35 (153)	32.7 (143)	29 (102)	28.2 (98)
Moderate dependent	13.7 (60)	8.2 (36)	5.9 (20)	3.5 (13)
Severe dependent	3.2 (14)	2.8 (12)	1.2 (4)	1.5 (4)
Total dependent	0.9 (4)	0.9 (4)	0.02 (1)	0.05 (2)
	348		276	
**Timed Up and Go**				
No risk of falls	53.2 (185)	66 (230)	65.5 (183)	74.8 (208)
Risk of falls	46.8 (163)	34 (118)	34.5 (93)	25.2 (68)
**Mini-Mental State Examination % (n)**	348		336	
No cognitive impairment	45.1 (157)	52.3 (182)	52.4 (176)	53 (178)
Mild cognitive impairments	27.9 (97)	22.7 (79)	24.1 (81)	23.8 (80)
Suspected dementia	27 (94)	25 (87)	23.5 (79)	23.2 (78)
**Geriatric Depression Scale-15 % (n)**	372		284	
No depression	46.8 (174)	53.5 (199)	57 (162)	63.4 (180)
Mild depression	23.4 (87)	26 (97)	22.2 (63)	20.4 (58)
Moderate/severe depression	29.8 (111)	20.5 (76)	20.8 (59)	16.2 (46)
**EQ-5D % (n)**	324		223	
Excellent/very good/good	53.0 (170)	62.5 (201)	54.8 (122)	58.3 (130)
Fair/poor	47.0 (154)	37.5 (123)	45.2 (101)	41.7 (93)

Values are expressed as percentages (%), with absolute frequencies shown in parentheses. This table complements Supplementary Figures 1 and 2 and presents the distribution of participants across functional status, mental health, and quality of life categories at entry and exit in 2022 and 2023.

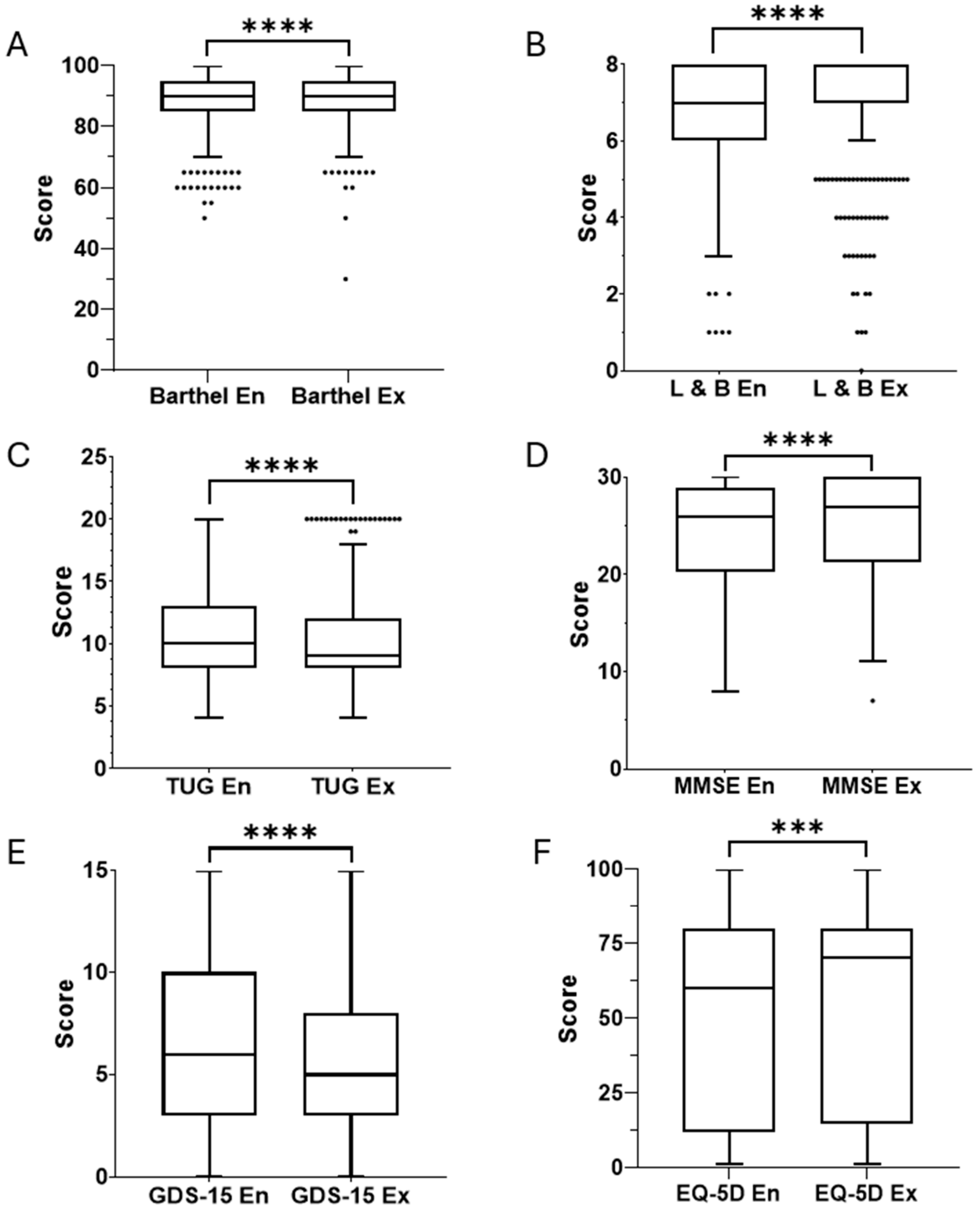

Figure S1. s. Boxplots showing entry (En) and exit (Ex) scores for functional status, mental health, and quality of life in 2022. Panels A–F correspond to: BI (A), L&B (B), TUG (C), MMSE (D), GDS-15 (E), and EQ-5D (F). Boxes represent IQRs, horizontal lines indicate medians, whiskers show 1.5×IQR, and dots are outliers. Comparisons used the Wilcoxon signed-rank test. *** p < 0.001; **** p < 0.0001.

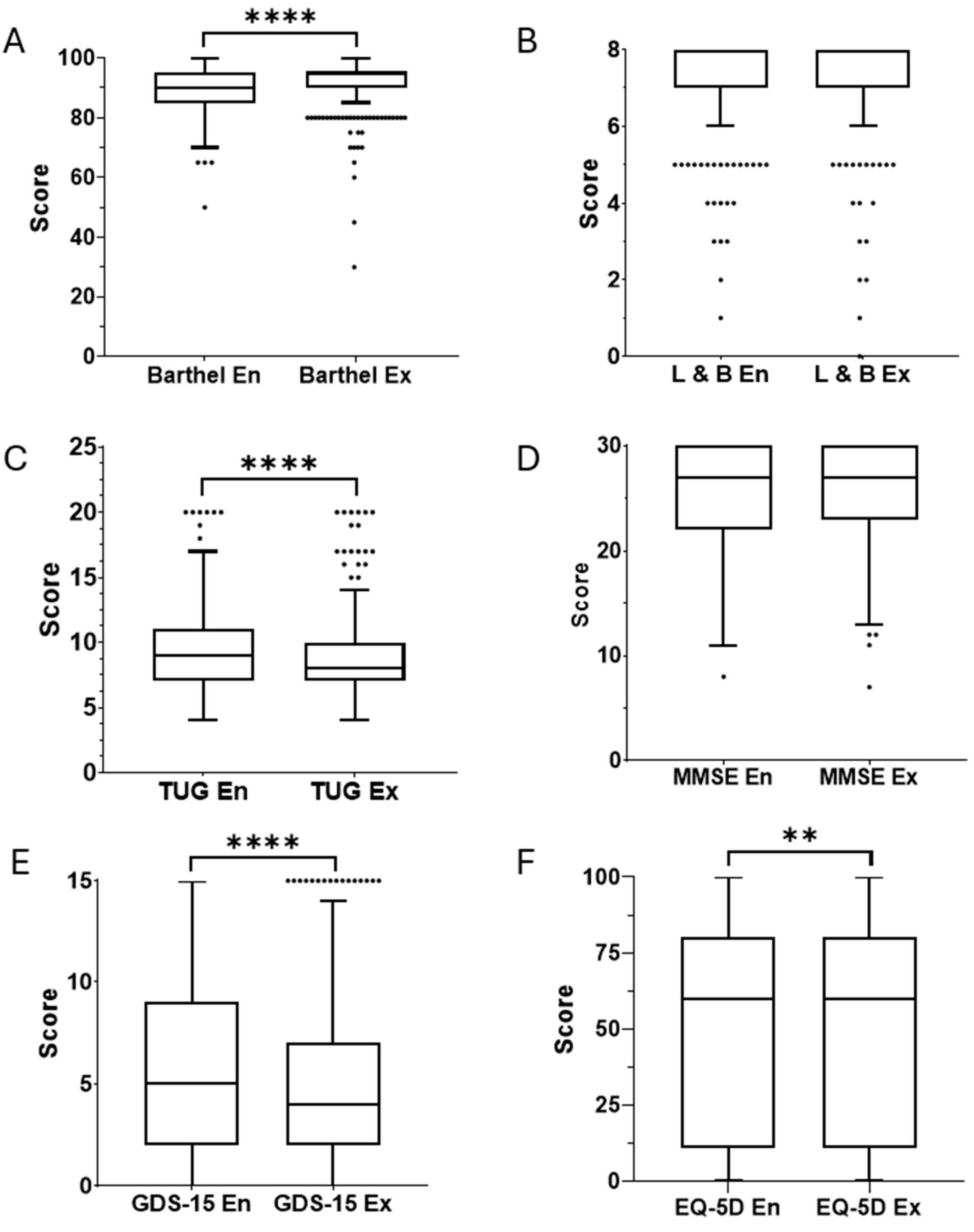

Figure S2. Boxplots showing entry (En) and exit (Ex) scores for functional status, mental health, and quality of life in 2023. Panels A–F correspond to: BI (A), L&B (B), TUG (C), MMSE (D), GDS-15 (E), and EQ-5D (F). Boxes represent IQRs, horizontal lines indicate medians, whiskers show 1.5×IQR, and dots are outliers. Comparisons used the Wilcoxon signed-rank test. ** $p < 0.01$; **** $p < 0.0001$.

.
